# 3D Printing of UV-Curable Polyurethane Incorporated with Surface-Grafted Nanocellulose

**DOI:** 10.3390/nano9121726

**Published:** 2019-12-03

**Authors:** Denesh Mohan, Mohd Shaiful Sajab, Hatika Kaco, Saiful Bahari Bakarudin, An’amt Mohamed Noor

**Affiliations:** 1 Research Center for Sustainable Process Technology (CESPRO), Faculty of Engineering and Built Environment, Universiti Kebangsaan Malaysia, Bangi 43600, Malaysia; denesh.mohan@gmail.com; 2 Department of Chemical & Process Engineering, Faculty of Engineering and Built Environment, Universiti Kebangsaan Malaysia, Bangi 43600, Malaysia; 3 Kolej GENIUS Insan, Universiti Sains Islam Malaysia, Bandar Baru Nilai 71800, Malaysia; hatikakaco@usim.edu.my; 4 Institute of Microengineering and Nanoelectronics (IMEN), Universiti Kebangsaan Malaysia, Bangi 43600, Malaysia; saifulbahari@ukm.edu.my; 5 Advanced Materials Research Cluster, Faculty of Bioengineering and Technology, Universiti Malaysia Kelantan, Jeli Kampus, Jeli 17600, Malaysia; anamt@umk.edu.my

**Keywords:** additive manufacturing, cellulose nanofibrils, nanocomposites, nanoindentation, stereolithography

## Abstract

The recognition of nanocellulose has been prominent in recent years as prospect materials, yet the ineffectiveness of nanocellulose to disperse in an organic solvent has restricted its utilization, especially as a reinforcement in polymer nanocomposite. In this study, cellulose has been isolated and defibrillated as cellulose nanofibrils (CNF) from oil palm empty fruit bunch (EFB) fibers. Subsequently, to enhance its compatibility with UV-curable polyurethane (PU)-based resin, the surface hydrophilicity of CNF has been tailored with polyethylene glycol (PEG), as well as reduced graphene oxide (rGO). The dispersibility of reinforced modified CNF in UV-curable PU was examined through the transmittance interruption of resin, chemical, and mechanical properties of the composite printed using the stereolithographic technique. Evidently, the enhanced compatibility of modified CNF and UV-curable PU was shown to improve the tensile strength and hardness of the composites by 37% and 129%, respectively.

## 1. Introduction

As the world’s second largest producer of palm oil products, Malaysia had an oil palm plantation area of 5.85 million hectares in 2017—an increase of 0.7% from the 5.81 million hectares in the previous year [1]. In tandem with the production of crude palm oil, the oil palm industry produces two forms of biomass waste, namely wastes from the mill and wastes from the field [2]. Even though large amounts of biomass are produced from the abovementioned agricultural waste, only 10% of the same is used as an alternative raw material in biocomposite-based industries, fertilizers, animal feeds, chemical derivatives, etc. [3]. It is estimated that in the mills, 23% of oil palm empty fruit bunches (EFB) are produced per ton of fresh fruit bunches [4]. Oil palm EFB is usually left as waste in the palm oil mill or used as boiler fuel for steam generation. Such use, however, has created an environmental pollution problem. Being rich in lignocellulose, there is huge potential for the production of nanocellulose, particularly for nanocomposite materials as reinforcing filler for the polymer industry, electronics, and also for biomedical purposes [5].

Oil palm EFB mainly consists of lignin, hemicelluloses, and cellulose, the last of which is the most abundant natural polymer worldwide [6]. The molecules of polymers like cellulose, hemicellulose, lignin, extractives, and pectin are responsible for the physical and mechanical properties of lignocellulosic materials [7]. Being a natural composite material, lignin binds the strands as well as the fibrils. Cellulose content and microfibrillar angle will determine the properties of the lignocellulosic fibers [8]. Therefore, the strengths of the fibers are dependent on the fibrillary structure, microfibrillar angle, and cellulose content [9]. Oil palm EFB fibers have sufficient strength and Young’s modulus to be used as biocomposites. As cellulose is also a carbohydrate polymer which consists of repeating units of β-D-glucopyranose joined together by β-1, 4-glycosidic linkage bonds, it possesses the suitable properties of the biodegradable polymer [10]. Up to now, the utilization of cellulose is still expanding from the conventional injection molding to the additive manufacturing of composites.

Despite the fused deposition modelling (FDM)-based technique being widely used due to the inexpensive machine and materials, the quality of surface finish and mechanical properties of these printed materials are limited in comparison with other additive manufacturing techniques and typical manufacturing processes [11,12]. Apart from this technique, stereolithography is the most common technique for the creation of a computer-designed virtual three-dimensional object to be “printed” into a solid object [13]. This technique typically used UV-curable resins consisting of oligomers, monomers (which act as diluents), photo-polymerization initiators, co-initiators (spectral sensitizers, reducing agents, etc.), and various additives such as stabilizers, antioxidants, plasticizers, as well as pigments [14]. The majority of commercial light-cured resins are based on free radical-cured acrylic compounds (acrylates), which constitute the most versatile curing systems concerning properties of the products (monomers/oligomers) available in the market. Meanwhile, the thermoset nature of stereolithography-fabricated parts, along with their high crosslink density, results in brittle fractures with poor elongation properties [15].

Cellulose to cellulose nanofibrils (CNF) show a promising natural fiber for the reinforcement of polymer composites [8,16,17]. However, the highly hygroscopic nature of nanocellulose requires water-dispersible polymer matrices. The poor ability of nanocellulose to disperse in organic solvents has led to the separation of the matrix and fiber phases in the polymer nanocomposites [16]. Although lignocellulose is compatible with the hydrophilicity–hydrophobicity of cellulose/hemicellulose and lignin in nature, this unique feature is unlikely to manifest after fractionation owing to slight physicochemical changes in the isolated cellulose [18]. However, with the proper surface modification of nanocellulose (e.g., acetylation, polymer grafting) when acetylated cellulose was added to nitrile butadiene rubber, the mechanical properties of the compatibilized nanocellulose can significantly improve polymer nanocomposite material [19]. Furthermore, the introduction of polyethylene glycol (PEG) as an interfacial agent specifically prevents the agglomeration and allows the dispersion of the CNF for a homogeneous structure [20]. Alternatively, the hydrophobicity of reduced graphene oxide (rGO) can also be used to cover the hydroxyl group of cellulose and disperse well with the hydrophobic solvent [21].

Thus, this study has attempted to determine the extent of improvement of the mechanical properties for UV-curable polyurethane (PU)-based 3D-printed products by enhancing the compatibility of CNF as a reinforced material in the resin composition. CNF, which has been isolated and defibrillated from oil palm biomass of EFB fibers, was modified with PEG and rGO as compatibilizer and surface modifier. In-depth analysis of the chemical interactions between CNF, PU resin, and the modified CNF was performed and correlated with the tensile strength, Young’s modulus, and the hardness of the printed composites.

## 2. Materials and Methods

### 2.1. Materials

Oil palm EFB fibers procured from Szetech Engineering Sdn Bhd (Selangor, Malaysia) were milled and sieved at desired sizes of 106 to 500 µm. Preparation of cellulose isolation and the defibrillation processes were done using formic acid, 90%, hydrogen peroxide, 30%, sodium hydroxide, and iron (II) sulfate heptahydrate received from Merck, Darmstadt, Germany. While the surface-grafted CNF were formulated using PEG with an average M_n_ of 4000 (Linear formula, H(OCH_2_CH_2_)nOH) (Sigma-Aldrich, Darmstadt, Germany), synthesized reduced graphene oxide with graphite flakes (Ashbury, Inc., Ruckersville, VA, USA), phosphoric acid, 85%, potassium permanganate, 99.9%, hydrogen peroxide, 30%, and sodium borohydride (Merck, Darmstadt, Germany). In 3D printing, commercial UV-curable resin was used with a composition of 45–47 wt% polyurethane acrylate, 34–36 wt% morpholine, and 15–17 wt% tripropylene glycol diacrylate (Wanhao Precision Casting Co. Ltd. (Jinhua, China)), and isopropyl alcohol (Merck, Darmstadt, Germany) was used to remove the residual resin on the 3D-printed samples.

### 2.2. Isolation and Defibrillation of Cellulose

The isolation of cellulose was performed in accordance with the protocol of a previous study [22,23]. In further detail, oil palm EFB were reacted with formic acid (90% w/v) at a 1:30 weight ratio at 95 °C for 2 h. The experiments were performed in three-necked flat-bottomed flasks equipped with a condenser. Using a digital hotplate magnetic stirrer, the reagents were stirred at 800 rpm (MSH-20D, Daihan Scientific, Gangwon-do, Korea). Subsequently, the supernatant and pulp were separated via vacuum filtration (MVP 10, IKA, Staufen, Germany). The pulp was then bleached with NaOH (2 wt%) and H_2_O_2_ (2 wt%) to degrade lignin and hemicellulose. Catalytic oxidation was performed to further purify to cellulosic pulp fraction. Specifically, a low concentration of H_2_O_2_ (2% w/v), along with 10 mg/L Fe(II), was added to the reaction mixture and incubated at 90 °C for 24 h. The chemical residues were then removed by thorough washing with deionized water. From there, the extracted cellulose was kept in a 4 °C refrigerator overnight. Next, the purity and yield of the cellulose were closely monitored according to the National Renewable Energy Laboratory (NREL) standard. Chemical analysis was then performed to further characterize the cellulose.

To yield CNF via mechanical shearing, 0.7 wt% of cellulose solution was fibrillated using a high-speed blender at 37,000 rpm (Vitamix 5200, Vitamix, OH, USA) for 30 min in the presence of a control cycle. The temperature was consistently maintained at 70 °C throughout the process to prevent the hydrolysis of cellulose.

### 2.3. Surface Modification of Cellulose Nanofibrils

PEG was used to reduce the hydrophilicity of the CNF in order to improve their dispersibility in the resin matrix. Specifically, 0.5 wt% of PEG 4000 was dissolved in ethanol solution and added to the CNF solution with reference to the CNF content, after which the mixture was homogenized using an homogenizer for 30 min (T 25 Ultra-Turrax, IKA, Staufen, Germany). In the preparation of rGO, graphene oxide (GO) was prepared via Hummers’ method with slight modifications. First, oxidation was carried out by mixing of H_2_SO_4_ (400 mL), graphite flakes (3 g), and KMnO_4_ (18 g), after which the mixture was stirred for 3 days using a magnetic stirrer to ensure the completion of graphite oxidation, as denoted by a color change from dark purplish-green to dark brown. Next, H_2_O_2_ (27 mL) solution was added to the mixture to terminate the oxidation. Concurrently, the color of the mixture changed to bright yellow, indicating that the GO was highly oxidized. The resulting GO was washed thrice with HCl solution (1 M) and ten times with deionized water until the pH of the product was in the range of 4 to 5. Washing was performed using a centrifugation technique, whereby the CNF solution was mixed with 10 wt% GO solution using homogenizer for 30 min (T 25 Ultra-Turrax, IKA, Staufen, Germany), after which NaBH_4_ was added to the CNF/GO mixture and heated at 80 °C for 60 min to yield CNF/rGO.

### 2.4. Resin Composition for Stereolithography

Commercial resin-based polyurethane was mixed vigorously with surface modified CNF with their respective proportions using homogenizer for 30 min (T 25 Ultra-Turrax, IKA, Staufen, Germany). As a reference, PU was homogenized with 0.005 wt% of PEG, equivalent to the PEG comprised in PU-1%CNG/PEG and 0.001 wt% of rGO, equivalent to the rGO comprised in PU-1%CNF/rGO. The mixture was then added to digital light-processing (DLP) 3D printer system (Duplicator D7 Plus, Wanhao, Jinhua, China), with a 405 nm UV lamp as a curing agent. The STL file model was followed by standard tensile specimen according to the ASTM D638 Type IV. The density was measured by the dimension and weight of printed samples. Meanwhile, each of the five printed layers was exposed for 70 s. The final product was then washed in an isopropyl alcohol solution to remove the excessive polyurethane resin prior to further cure under UV light of wavelength 405 nm. Meanwhile, the kinetics of the curing process was determined in terms of the weight of solid formed per minute. Briefly, 7 g of the resin with modified CNFs was added in the 3D-printed mold with similar dimensions to the standard tensile specimen and was exposed with a 405 nm UV lamp on the top of the 3D printer projector. The cured specimen at a determined time, *t*, was washed with isopropyl alcohol solution and the weight was recorded.

### 2.5. Characterization

The functional groups were characterized using Fourier transform infrared spectroscopy (FTIR, Bruker, Billerica, MA, USA) at a resolution of 1 cm^−1^ in a wavelength range of 650–4000 cm^−1^. In addition, CNF, CNF/PEG, CNF/rGO, and GO had their morphological structures analyzed via transmission electron microscopy (TEM) (CM12 Philips, Eindhoven, Netherlands). The crystalline regions of the samples were evaluated via X-ray diffractometry (XRD) (Bruker D8 Advance, Bruker, Billerica, MA, USA). Furthermore, the transmittances of the PEG and rGO-modified polyurethane resins were measured using an SP-300SRB UV Spectrophotometer. Thermal properties of the samples were measured using a differential scanning calorimeter (Shimadzu/DSC50, Japan) under a nitrogen atmosphere. The viscosity of the resin was examine using Brookefield Ametek D1 viscometer with spindle type DV1HA at 100 rpm. Samples (5 mg) were hermetically sealed in an aluminum pan and a sealed empty pan was used as the reference. Samples were first heated to 300 °C at a heating rate of 10 °C/min. On another note, the tensile strength and Young’s modulus of the samples were determined using Instron® Electromechanical Universal Testing Systems 3300 Series at 500 mm/min with a load cell of 1 kN. The high loading rates was chosen based on the reproducibility of results for tensile strength and compliance with the previous study [24,25]. The micrograph of the cross-section 3D-printed samples after tensile testing was observed by a field emission scanning electron microscope, FESEM (Merlin Compact, Zeiss Pvt Ltd., Oberkochen, Germany), and the element mapping was monitored by energy dispersive X-ray analysis, EDX (Oxford Instruments GmbH, Wiesbaden, Germany). Finally, the nanoindentation behaviors of the materials were determined using a Nano TestTM (Micro Materials, Wrexham, UK) with a maximum load of 5 mN. Five times for each parameter were repeated tensile test and nanoindentation analyses.

## 3. Results and Discussion

### 3.1. Characterization of Surface-Grafted CNF

**Figure 1:**
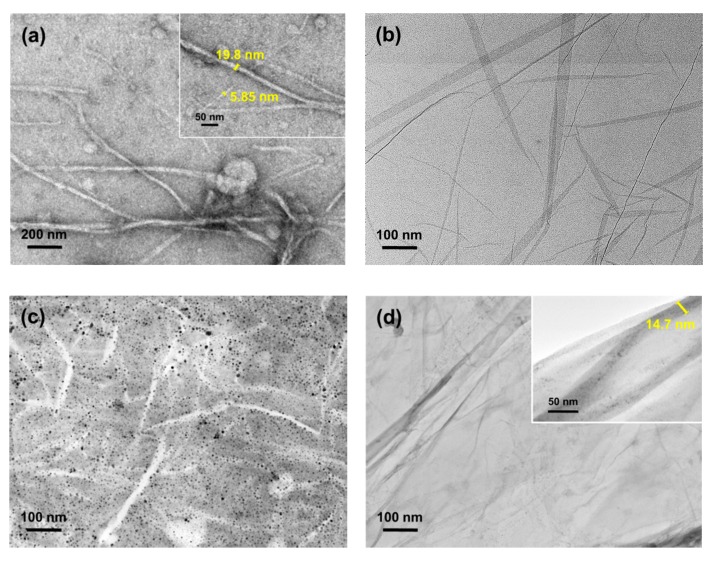
The micrograph of (**a**) unmodified cellulose nanofibrils (CNF), (**b**) reduced graphene oxide (rGO), (**c**) surface-grafted CNF/polyethylene glycol (PEG), and (**d**) surface-grafted CNF/rGO.

Figure 1a shows the successful cellulose defibrillation, whereas the individual fibrils were measured at comparable diameters average of 5 to 20 nm [26]. Contrastingly, GO in Figure 1b appeared as a semi-transparent sheet and looks unstable when morphology was observed in TEM. The disordered and unwrinkled structure was attributed to the non-removal of oxygen atoms and a high degree of exfoliation during oxidation [27]. The micrograph of CNF/PEG shown in Figure 1c presents the dispersibility of CNF with the PEG, while the trace of CNF under a thin layer of rGO was visibly present in Figure 1d [28]. Moreover, the modified CNF shows both the stability of interfacial agents and remains as individual fibrils.

**Figure 2:**
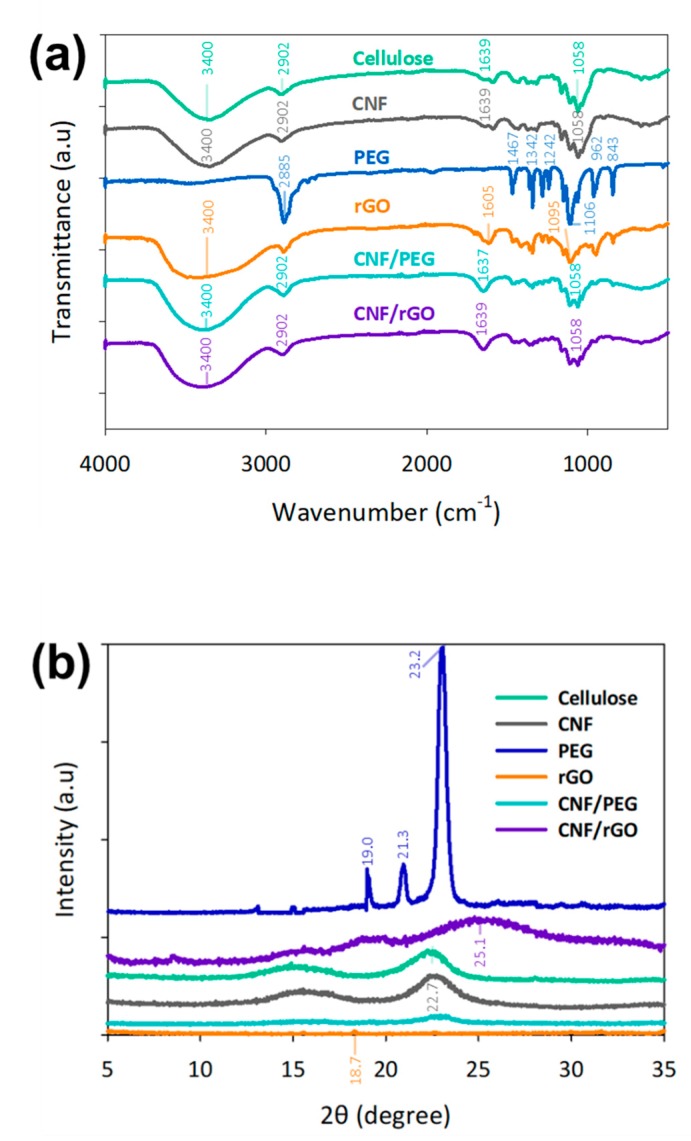
Chemical characterization of isolated cellulose, unmodified CNF, PEG, rGO, and surface-modified CNF/PEG and CNF/rGO on (**a**) Fourier transform infrared spectroscopy (FTIR) and (**b**) X-ray diffractometry (XRD) spectrum.

In the chemical characteristic of isolated cellulose and CNF, Figure 2a indicates the absorption peaks at 3400 cm^−1^ due to the presence of hydroxyl groups and stretching of the saturated aliphatic C-H of lignin and cellulose. These results were generally in line with those of a recent study on cellulose derivatives [29]. Likewise, the absorption peaks around 2902 and 1058 cm^−1^ were attributed to the C-H and C-O stretching vibrations on cellulose and CNF respectively. Meanwhile, the significant peaks of PEG were observed around 2885 cm^−1^ due to aliphatic C-H stretching, at 1106 cm^−1^ due to C-O stretching, and at 1467, 1342, 962, 843 cm^−1^ attributed to the C-H vibration in the PEG skeleton [30]. FTIR spectra of rGO had absorption peaks at around 1605 and 1095 cm^−1^ due to recovery of sp^2^ lattice and C-O stretching respectively [31]. Additionally, CNF/rGO and CNF/PEG had characteristic absorption peaks, which were the same as those of CNF. This could be explained by the hypothesis that cellulose nanofibrils were merely combined with rGO and PEG by physical interactions without the formation of new functional groups.

As shown in Figure 2b, the similarities of the patterns between both CNF and cellulose were maintained after the mechanical shearing. The crystallinity index increases after the treatment from 62.93% to 75.03% [32]. PEG 4000 showed sharp diffraction peaks at 2θ of 19°, 21.3°, and 23.2°, thereby indicating the presence of crystalline regions. After the chemical reduction of GO to rGO, the ordered crystal structure of rGO was restored as a peak was observed at 18.7° [33]. The weak and broad diffraction peak of rGO exhibits the interlayer spacing between graphene and restoration of graphitic structure after the chemical reduction [34]. CNF/PEG has a peak at 23.2° owing to the presence of PEG, while CNF/rGO has a peak of around 25.1°, which indicates the reduction of oxygen-containing functional groups in GO structures [35].

### 3.2. Transmittance and Curing Efficiency of Resin Composition

**Figure 3:**
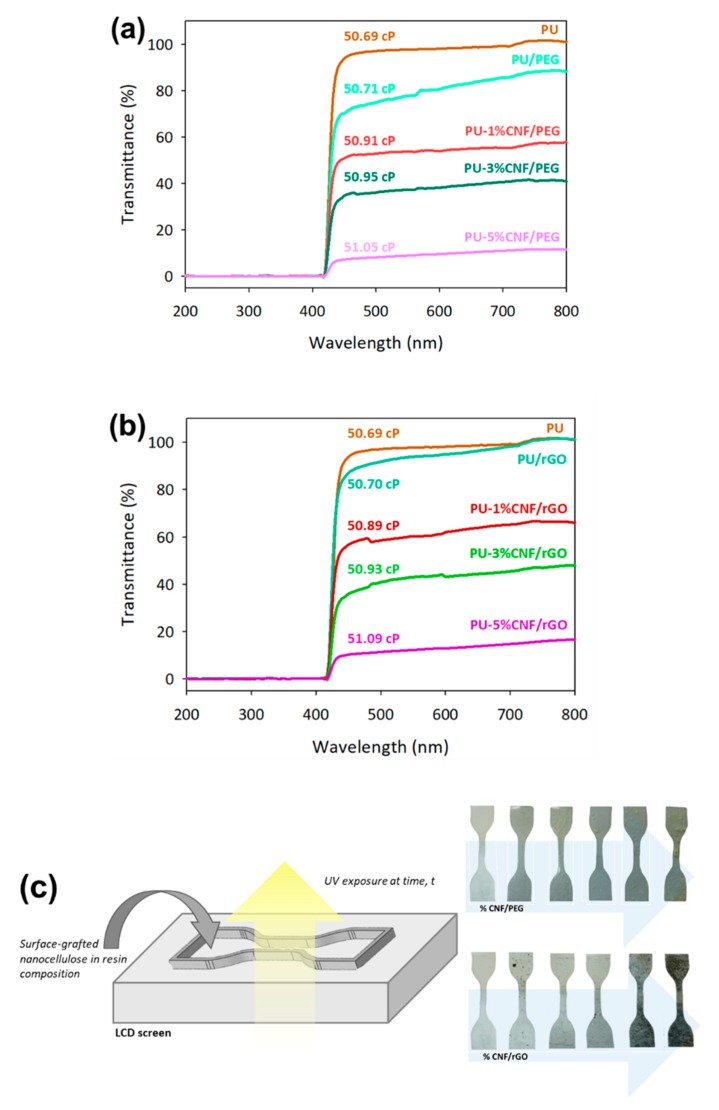
The transmittance of UV-curable polyurethane (PU)-based resin with the surface-grafted CNF composition of (**a**) CNF/PEG, (**b**) CNF/rGO, and (**c**) the curing process exposed in the 3D printer projector.

Figure 3 shows the transmittance interruption at 405 nm, which is attributable to the specific peak of polymerization for UV-curable PU resin [12]. The average transmittance of PU/rGO was higher than that of PU/PEG, which remains uninterrupted for the curing and polymerization process due to the thin layer of rGO shown in Figure 1d. However, the presence of CNF in the resin composition reduced the transmittance of selected wavelength significantly as the unmodified CNF decrease light transmission [26]. In addition, the effect of transmittance interruption of surface-grafted CNF on the curing efficiency of the resin composition was observed by UV exposure time on the top of the DLP projector (see Figure 3c). As a result, the PU resin, which contained a CNF modified rGO, was producing a higher solid formed per minute as a comparison with CNF modified PEG (see Table 1). The amount of PU-3%CNF/rGO product generated (in terms of weight) was higher than that of PU resin despite the former having a lower absorbance. This phenomenon was due to the higher density of CNF relative to PU resin. Meanwhile, the amount of PU-5%CNF/PEG produced was lower owing to its interruption of the transmitted wavelength.

As a side note, the mixture of PU-based commercial resin with CNF exhibits a thick agglomeration with the inhomogeneous composition. The water moisture present in unmodified CNF on account of the hygroscopic nature of cellulose intervenes in the interface polymerization with hydrophobic polymers matrix such as PU [8,36]. In the addition of PEG and rGO as a compatibilizer, modified CNF was able to distribute well in the PU matrix. Hydrogen bonds could be formed between the PU matrix and CNF owing to the presence of PEG [20], whereby rGO layers acted as a moisture barrier which enabled the hydrophilic CNF particles to disperse well in the PU matrix without aggregates being formed [27,37]. Although the addition of surface-grafted CNF increased the viscosity of the resin, however, the tabulated data in Figure 3a,b provided low significant changes (~2.4%) even at higher concentration of CNFs added in the resin.

### 3.3. Characterization of UV-Cured Composites

**Figure 4:**
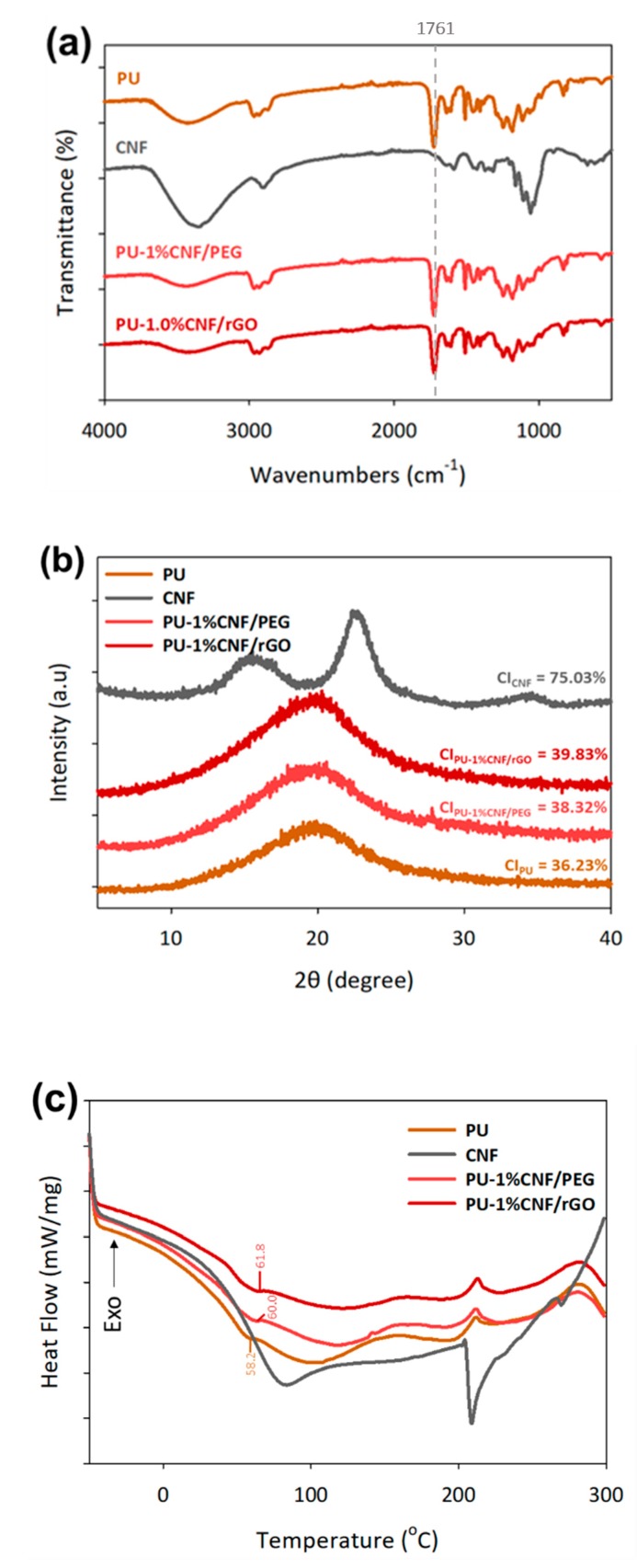
Characterization and comparison between the UV-cured composites and unmodified CNF and PU resin on (**a**) FTIR, (**b**) XRD, and (**c**) differential scanning calorimetry (DSC).

The chemical characterization of UV-cured composites was examined using FTIR, XRD, and DSC analyses, whereby the unmodified CNF and PU resin were used as reference materials (see Figure 4). In the FTIR spectrum, significant characteristic peaks of PU were still dominant, indicating there was no chemical interaction with polymer matrices upon addition of CNF/PEG and CNF/rGO, which highlighted the unaffected C = O of polyurethane peak at 1761 cm^−1^. Theoretically, the C-O-C and terminal -OH of PEG were able to form hydrogen bonds or dipolar interactions with PU and CNF, connecting the polymeric linkage like a bridge [38].

In the XRD patterns, PU resin displayed only one diffuse and amorphous halo, indicating that no well-defined ordered structures existed. The XRD pattern intensity increases with the addition of 1% of CNF/PEG and CNF/rGO as the implementation of CNF disrupts the original uniform and makes the PU matrix more crystalline. The DSC plots in Figure 4c show the glass transition temperatures (T_g_) for PU, PU-1%CNF/PEG, PU-1%CNF/rGO are 58.2, 60.0, and 61.8 °C, respectively. This represents the temperature range in which the polymer substrate shifts from a stiff glass material to a smooth material [39]. The T_g_ increases with the addition of surface-grafted CNF, which can be attributed to the fact that, as stated earlier, the crystallinity of the PU/CNF composite increased with the addition of CNF, resulting in higher stiffness and rigidity of the composite [40].

### 3.4. Mechanical Properties

**Figure 5:**
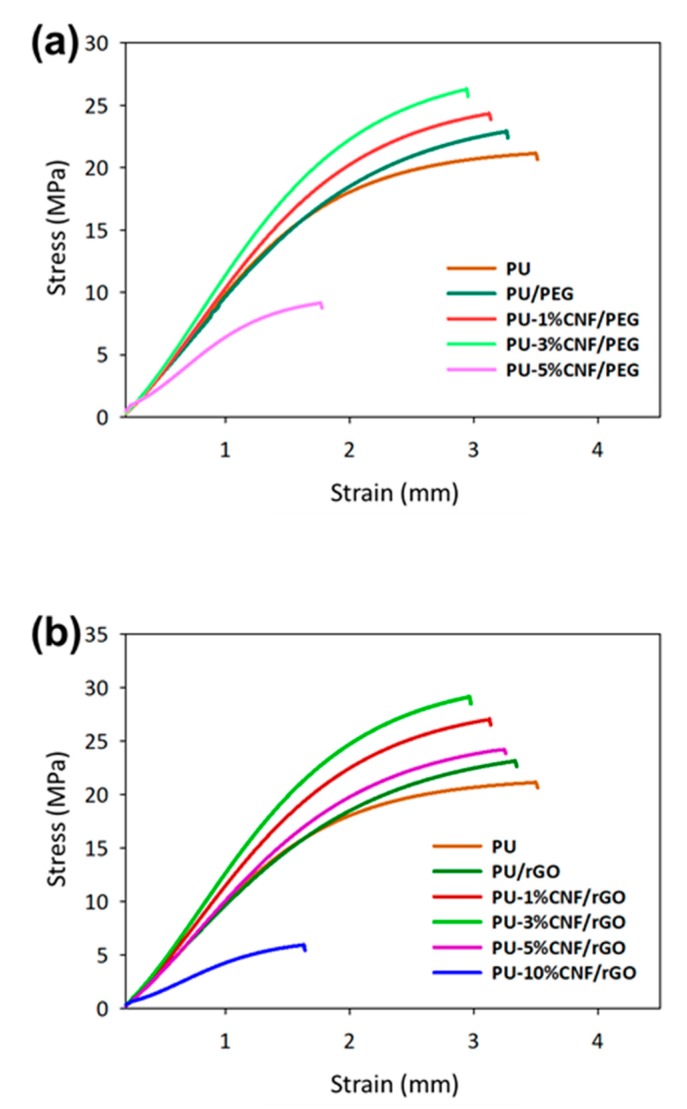
Stress–Strain curve of UV-cured PU and reinforced modified CNF with (**a**) PEG and (**b**) rGO.

The dispersibility of the reinforced modified cellulose in UV-cured PU was investigated using tensile properties of the materials shown in Figure 5. As a reference, the addition of PEG and rGO to PU (wt% equivalent of PEG and rGO contained in 1% of modified CNF) improved the tensile strength by 8.3% and 9.37%, respectively. This could be attributed to the fact that the CNF/PEG and CNF/rGO facilitated the crystallization process of PU [41]. Alternatively, graphene is the polymer nanofiller of choice because its layered structural fillers have a large surface area (up to 2630 m^2^/g) [42]. Thus, a lower loading of graphene improved the properties of the composite owing to its high aspect ratio [43].

Therefore, PU-3%-CNF/PEG showed a 24% increment in its tensile strength relative to PU. Accordingly, the tensile strength of PU-3%CNF/rGO was 37% and 11% better than those of PU and 3%-CNF/PEG. Although the presence of PEG facilitated the interactions between the hydrophobic polymer and the hydrophilic CNF, rGO provided better removal hydrophilicity of CNF than PEG due to the higher density of carbon atoms on its surface [20,44]. rGO can nullify the natural hydrophilicity of CNF, thereby enabling the latter to disperse well in hydrophobic PU [45]. Likewise, previous studies noted an improvement in the mechanical properties of CNF-composites following the addition of CNFs of concentrations below 3 wt% [46]. Meanwhile, the loading of 5% modified CNF resulted in a decline in the tensile properties due to the generation of non-uniform stress transfer when the sample was under tension loading. The reason for this finding was when the filler loading exceeded 5 wt%, the CNF particles started to aggregate in the polymer matrix and introduce more interfacial voids which reduced the tensile strength of the composite [47]. As a consequence, the entire nanocomposite could have acted as a stress concentrator and hence, the mechanical properties declined [48]. Moreover, a higher CNF loading interrupted the curing of the photocurable PU resin and the printed samples orientation is affected due to insufficient curing at higher CNF loading rate.

**Figure 6:**
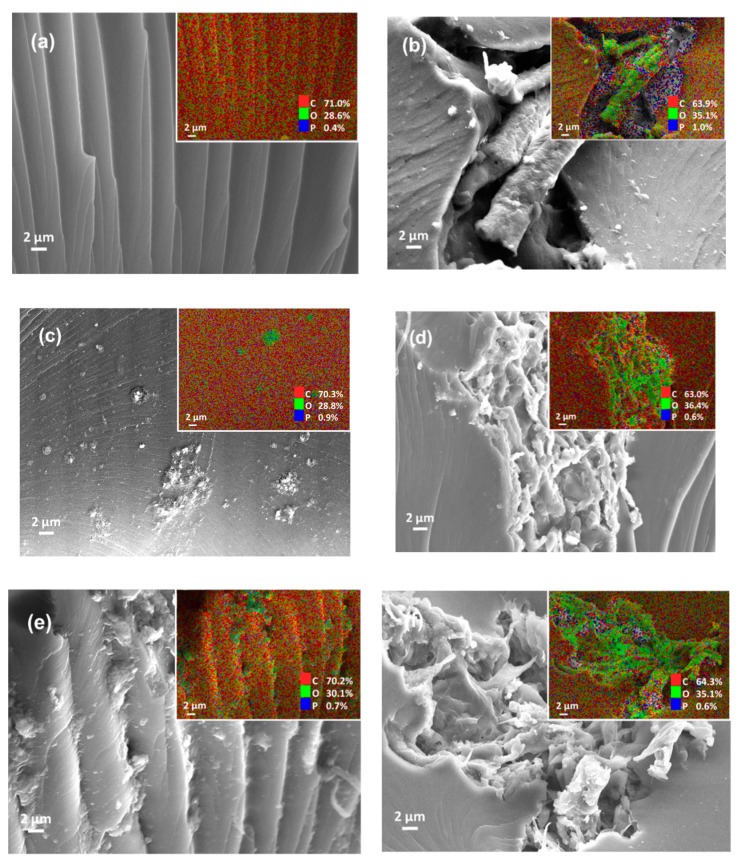
The micrograph and energy dispersive X-ray analysis (EDX) mapping of cross-section 3D-printed samples after tensile testing (**a**) PU, (**b**) PU-3%CNF, (**c**) PU/PEG, (**d**) PU-3%CNF/PEG, (**e**) PU/rGO, and (**f**) PU-3%CNF/rGO.

The tensile strength of the UV-cured composites was correlated with Young’s modulus materials (see Table 2). Evidently, the addition of CNF/PEG and CNF/rGO improved the Young’s modulus of the composite. However, a higher loading of filler-reinforcer (i.e., PU-5%CNF/rGO) often resulted in particle agglomeration and, hence, a decline in Young’s modulus. As per previous studies, Young’s modulus of the PU improved 7-fold following the incorporation of 4 wt% GO [49]. The incorporation of rGO instead of PEG in CNF enhanced the mechanical properties of the composite since rGO better nullified the hydrophilicity of CNF, subsequently improving the dispersion and mechanical properties. The dispersibility of the modified CNF in the 3D-printed samples was supported with the morphological structure of cross-section 3D-printed samples after tensile testing and the elements mapping shows in Figure 6. The neat PU-based resin of 3D-printed samples shows a clean wide line and smooth surface with the composition of carbon, oxygen, and phosphorus. In the addition of CNF, the oxygen composition increased owing to the rich hydroxyl groups of cellulose [26]. The mixture of PU and unmodified CNF shows a visible void of interfacial polymerization due to the large hydrophilic surface at the sites of cellulose chain and hydrophobic polymers matrix of PU (see Figure 6b) [8,36]. On the other hand, the modified CNF provided better dispersibility between PU and CNF/PEG and CNF/rGO. The reinforcement of fibrillated cellulose can be obviously seen in the fracture of the composites.

**Figure 7:**
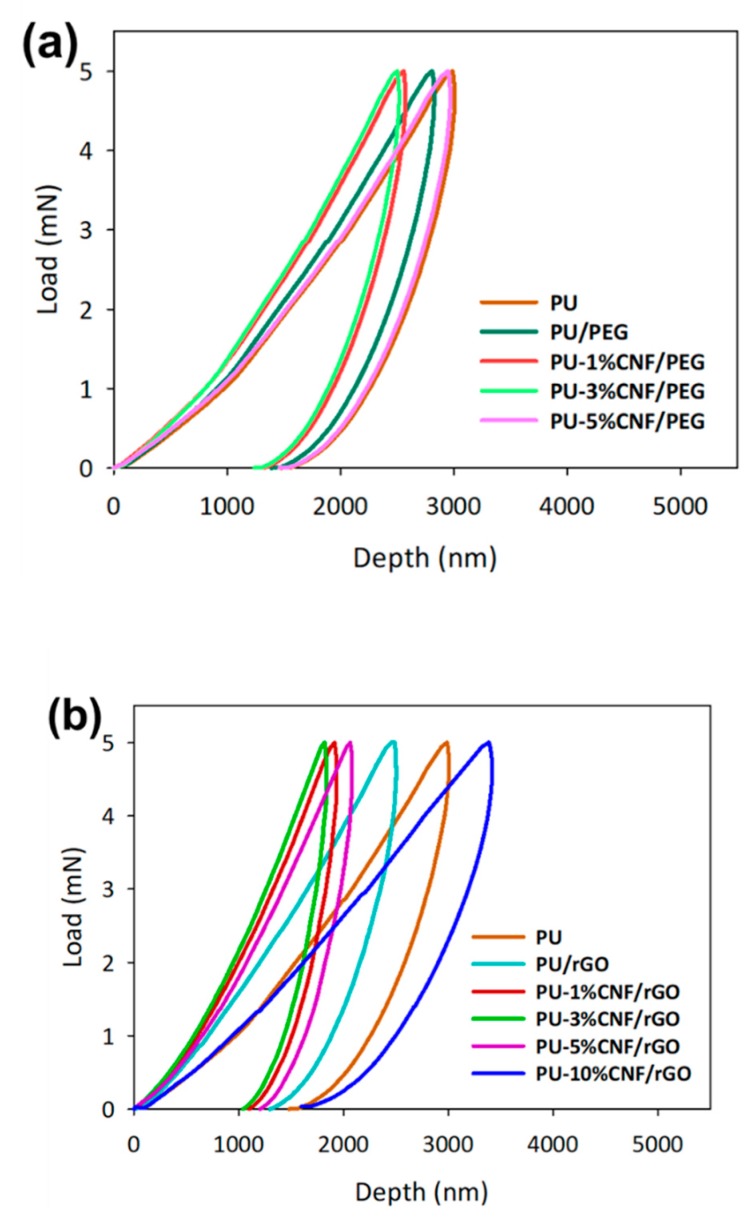
Nanoindentation displacement for UV-cured PU and reinforced with modified CNF with (**a**) PEG and (**b**) rGO.

On another matter, the UV-cured composites have been analyzed by nanoindentation with a maximum load of 5 mN. The loading–unloading curves of the sample were presented in Figure 7. Following the addition of CNF/PEG and CNF/rGO, the loading-unloading pattern showed a typical trend, unlike the stress–strain curve. Meanwhile, the depth of indentation decreased, with an increase in hardness. Concurrently, the curves were shifted to the left, indicating an improvement in hardness trend when the CNF percentage was increased [50]. With a hardness of 75.04 MPa (i.e., a 129% increment from unmodified UV-cured PU), PU-3%-CNF/rGO showed the greatest improvement in this parameter. Apparently, the enhancements of the hardness and elastic modulus of CNF/rGO-reinforced PU composites could have been due to the presence of strong interactions and bonds between the functional groups of rGO and the PU chains [51]. Furthermore, the hydrophilicity of CNF was better removed in the presence of rGO. The basis for this occurrence was that rGO was a better plasticizer than PEG, so it better facilitated interactions with PU. However, when the concentration of graphene was increased to 3%, a trend which was similar to that of the stress–strain curve (i.e., a decline in hardness with an increase in loading) interrupted the curing behavior of the UV-cured PU and, hence, gave rise to defects in the orientation of the printed samples.

## 4. Conclusions

In this study, the dispersibility of modified CNF was exhibited through the enhanced mechanical properties, as the tensile strengths of PU-3%CNF/rGO and PU-3%CNF/PEG were 37% and 24% higher than UV-cured PU, respectively. Meanwhile, as per the outcomes of nanoindentations, PU-3%CNF/rGO had a hardness of 75.04 MPa—a 129% increase relative to that of unmodified photocurable PU. Likewise, PU-3%CNF/PEG had an 82% increase in its hardness. However, the increment of CNF (more than 3 wt%) showed aggregation in the polymer matrix and introduces more interfacial voids, which reduced the mechanical properties of the composites.

## Figures and Tables

**Table 1:** The kinetics of the curing process exposed in the 3D printer projector by the weight of solid formed per minute.

Time, *t* (min)	Weight of Sample After Curing at a Time, *t* (g)								
	PU	PU/PEG				PU/rGO			
		0% CNF	1% CNF	3% CNF	5% CNF	0% CNF	1% CNF	3% CNF	5% CNF
1	1.08	1.08	1.09	1.14	1.06	1.11	1.14	1.21	1.10
2	1.85	1.86	1.89	1.94	1.77	1.91	1.99	2.08	1.82
3	2.06	2.07	2.16	2.21	1.96	2.12	2.27	2.36	2.01
4	2.21	2.22	2.30	2.35	2.14	2.28	2.42	2.51	2.20
5	2.36	2.37	2.41	2.46	2.30	2.43	2.53	2.63	2.37
6	2.46	2.47	2.50	2.55	2.40	2.53	2.63	2.73	2.47
7	2.52	2.53	2.56	2.61	2.46	2.60	2.69	2.79	2.53
8	2.52	2.53	2.56	2.61	2.46	2.60	2.69	2.79	2.53
9	2.52	2.53	2.56	2.61	2.46	2.60	2.69	2.79	2.53

**Table 2:** Young’s modulus and loading–unloading behavior of UV-cured PU and reinforced with modified CNF with PEG and rGO.

Sample	Tensile Strength (MPa)	Elongation (mm)	Young’s Modulus (MPa)	Max Depth (nm)	Hardness (MPa)
PU	21.24 ± 0.21	3.50 ± 0.21	9.79 ± 0.15	3008 ± 15	32.7 ± 0.2
PU-PEG	22.93 ± 0.20	3.26 ± 0.14	9.74 ± 0.12	2538 ± 3	40.2 ± 0.2
PU-1%CNF/PEG	24.34 ± 0.19	3.14 ± 0.15	10.15 ± 0.12	2229 ± 11	52.8 ± 0.1
PU-3%CNF/PEG	26.30 ± 0.23	2.94 ± 0.19	10.78 ± 0.14	2185 ± 7	59.6 ± 0.3
PU-5%CNF/PEG	9.14 ± 0.16	1.76 ± 0.23	6.18 ± 0.09	2707 ± 5	32.5 ± 0.1
PU-rGO	23.15 ± 0.19	3.35 ± 0.23	10.43 ± 0.13	2502 ± 8	46.9 ± 0.2
PU-1%CNF/rGO	27.07 ± 0.30	3.14 ± 0.15	10.88 ± 0.16	2000 ± 12	74.4 ± 0.3
PU-3%CNF/rGO	29.16 ± 0.26	2.97 ± 0.21	12.41 ± 0.18	1934 ± 8	75.0 ± 0.2
PU-5%CNF/rGO	24.21 ± 0.21	3.25 ± 0.24	9.93 ± 0.12	2018 ± 6	68.2 ± 0.3
PU-10%CNF/rGO	5.94 ± 0.12	1.64 ± 0.32	3.44 ± 0.09	3419 ± 7	26.4 ± 0.1
